# A rule-based model of insulin signalling pathway

**DOI:** 10.1186/s12918-016-0281-4

**Published:** 2016-06-01

**Authors:** Barbara Di Camillo, Azzurra Carlon, Federica Eduati, Gianna Maria Toffolo

**Affiliations:** Department of Information Engineering, University of Padova, Via Gradenigo 6A, Padova, 35131 Italy; Magnetic Resonance Center (CERM) and Department of Chemistry “Ugo Schiff”, University of Florence, Florence, Italy; European Molecular Biology Laboratory, European Bioinformatics Institute (EMBL-EBI), Wellcome Trust Genome Campus, Cambridge, UK

**Keywords:** Insulin signalling pathway, Rule-based modelling, Parametric sensitivity analysis, System robustness

## Abstract

**Background:**

The insulin signalling pathway (ISP) is an important biochemical pathway, which regulates some fundamental biological functions such as glucose and lipid metabolism, protein synthesis, cell proliferation, cell differentiation and apoptosis. In the last years, different mathematical models based on ordinary differential equations have been proposed in the literature to describe specific features of the ISP, thus providing a description of the behaviour of the system and its emerging properties. However, protein-protein interactions potentially generate a multiplicity of distinct chemical species, an issue referred to as “combinatorial complexity”, which results in defining a high number of state variables equal to the number of possible protein modifications. This often leads to complex, error prone and difficult to handle model definitions.

**Results:**

In this work, we present a comprehensive model of the ISP, which integrates three models previously available in the literature by using the rule-based modelling (RBM) approach. RBM allows for a simple description of a number of signalling pathway characteristics, such as the phosphorylation of signalling proteins at multiple sites with different effects, the simultaneous interaction of many molecules of the signalling pathways with several binding partners, and the information about subcellular localization where reactions take place. Thanks to its modularity, it also allows an easy integration of different pathways.

After RBM specification, we simulated the dynamic behaviour of the ISP model and validated it using experimental data. We the examined the predicted profiles of all the active species and clustered them in four clusters according to their dynamic behaviour. Finally, we used parametric sensitivity analysis to show the role of negative feedback loops in controlling the robustness of the system.

**Conclusions:**

The presented ISP model is a powerful tool for data simulation and can be used in combination with experimental approaches to guide the experimental design. The model is available at http://sysbiobig.dei.unipd.it/ was submitted to Biomodels Database (https://www.ebi.ac.uk/biomodels-main/# MODEL 1604100005).

**Electronic supplementary material:**

The online version of this article (doi:10.1186/s12918-016-0281-4) contains supplementary material, which is available to authorized users.

## Background

Biological systems, such as cell signalling pathways, act sensing input stimuli (e.g. extracellular ligands), and transmitting, processing and integrating this information to provide output signals able to regulate many essential cellular activities. Alterations in such information processing capability play a crucial role in the development of a disease state in many cell types [1,2].

Mathematical models are often used to understand the functioning of biological systems, thus providing a description of the behaviour of the system and allowing useful analysis of its emerging properties [3]. Moreover, mathematical models are powerful tools for data simulation and can be used in combinations with experimental approaches to guide the experimental design, whereas data collected from experiments may help validating and refining the computational models [4].

Signalling pathways are usually modelled using ordinary differential equations (ODEs), able to quantitatively describe the dynamics of the chemical species, in terms of mass action law and kinetic rate constants related to the speed of the chemical reactions occurring among the interacting species [5]. As a simpler alternative, logic-based models [6] have been also used. A detailed description of a signalling pathway requires including site-specific details of protein-protein interactions, since signalling proteins contain multiple functional domains and several sites of post-translational modifications. However, protein-protein interactions potentially generate a multiplicity of distinct chemical species, an issue referred to as “combinatorial complexity” [7]. Adopting ODEs to represent such complexity would require using a number of state variables equal to the number of possible protein modifications, thus making model specification tedious and error-prone.

In order to deal with the combinatorial complexity of signalling pathways, a novel approach for model specification known as Rule-based Modelling (RBM), has been introduced in the past years, able to deal with the description of all molecular interactions in a more efficient and compact way [8–10]. RBM is based on the key assumption of “modularity”, i.e. the assumption that molecular interactions only depend on local properties of the proteins [11,12]. According to this assumption, classes of reactions involving the same components are defined by means of a “rule” and are associated with the same rate law. Models specified using RBM can then be used for simulation using a number of different population-based or particle-based methods [13]. In particular, the rules specified can be used to automatically generate a system of ODEs, which can then be simulated [10].

An impressive example of the power of RBM is represented by the specification of the epidermal growth factor (EGF) receptor signalling, in which the interaction among 10^23^ distinct chemical species was codified by the use of only 70 rules [14]. As a more recent example, the specification of the complex ERBB receptor signalling network required 544 rules accounting for 18 proteins, over 30 protein domains, several linear motifs, and 56 sites of lipid and protein phosphorylation [12].

An important advantage of RBM resides in its modularity, which facilitates the integration of different pathways, as well as the model extension, when new knowledge becomes available.

In this work, we present a model of the insulin signalling pathway (ISP), based on RBM. ISP is one of the most important signalling network, which regulates some fundamental biological functions such as glucose and lipid metabolism, protein synthesis, cell proliferation, cell differentiation and apoptosis. These different biological responses are achieved by the insulin binding to its receptor and by the subsequent combined activation of three major pathways:

(i). The PI3K-AKT pathway, mostly responsible for the metabolic insulin action via the translocation of the glucose transporter type 4 (GLUT4) vesicles to the plasma membrane, which, in turn, allow the glucose uptake in muscle cells and adipocytes [15];
(ii). The TSC1/2-mTOR pathway, playing a critical role in protein synthesis since mammalian target of rapamycin (mTOR) is a central controller for several anabolic and catabolic processes including RNA translation, ribosome biogenesis and autophagy, in response not only to growth factors and hormones like insulin, but also to nutrients, energy and stress signals [16];
(iii). The RAS-MAPK pathway, promoting cell survival, division and motility via extracellular signal-regulated kinase 1/2 (ERK1/2) complex that, once phosphorylated, translocates into the nucleus activating many transcription factors, thus constituting an important connection between the cytoplasmic and nuclear events [17].

Several mathematical models of ISP have been published in the last 10 years, focused on different aspects of insulin regulation, including: insulin binding to its receptor [18]; insulin receptor autophosphorylation and subsequent phosphorylation of its substrate together with receptor cycling and endocytosis [19,20]; insulin signalling in insulin resistance state in human adipocytes [21]; translocation of GLUT4 glucose transporter [22]; regulation of mTOR [23,24]; joint regulation of insulin and amino acids [25]; crosstalk with epidermal growth factor (EGF) signalling and the mitogen-activated protein kinase (MAPK) pathway [26].

In this work, we adapted and integrated the information provided by three published models [22,24,26] to implement, up-to our knowledge, the most comprehensive model of the ISP. The three above listed models describe, using ODEs, the PI3K-AKT, the mTOR and the RAS-MAPK pathway, respectively, thus covering most of the essential regulatory mechanisms characterizing the ISP. The RBM approach was used to implement our ISP model. This was then partially validated by comparing the model predictions with measurements of some phosphorylated proteins involved in ISP, such as pAKT-S473, ppERK1/2-T202-Y204 and pmTOR-S2448. The model was then used as an in silico tool to predict the profiles of all the chemical species during insulin perturbation and to analyse, by means of parametric sensitivity analysis [27,28], the role of negative feedback loops in controlling the robustness of the system.

## Results and discussion

### The model of insulin signalling pathway

**Fig. 1:**
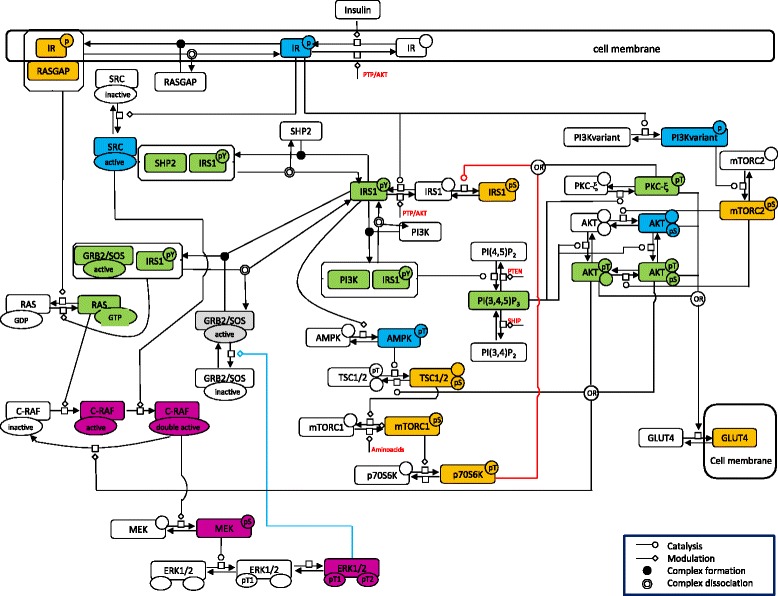
The model of insulin signalling pathway. The model of Insulin Signaling Pathway obtained by integrating the PI3K-AKT pathway, the mTOR pathway and the RAS-MAPK pathway, depicted using the Systems Biology Graphical Notation. Coloured nodes resemble the clustering results obtained on simulated profiles (see Fig. **3**). Coloured lines represent important feedback mechanisms; namely: the red line represents the P70S6K-IRS1 negative feedback loop, the blue line the ERK1/2-GRB2/SOS negative feedback loop

Starting from three published models [22,24,26], we implemented the model of ISP as depicted in Fig. 1 using the Systems Biology Graphical Notation [29].

The model comprises many of the essential elements responsible for the insulin action since the three major sub-pathways of the ISP, briefly described in the following, are included.

#### The PI3K-AKT pathway

For the PI3K-Akt pathway, we mostly refer to the model in Sedaghat et al. [22]. The model has been used by several research groups and includes many of the most known signalling components mediating the translocation of glucose transporter GLUT4. These include the insulin receptor binding and recycle subsystems; the post-receptor signalling subsystem including both Ser- and Tyr- phosphorylation at the insulin receptor substrate1 (IRS1); the formation of a complex (IRS1_PI3K complex) between phosphorylated IRS1 and phosphatidylinositide 3-kinase (PI3K); the phosphatidylinositol 3,4,5-trisphosphates PI(3,4,5)P_3,_ synthesis; the phosphorylation of protein kinase B (AKT) and protein kinase C (PKC)-ζ; the translocation of GLUT4 to the plasma membrane. Protein tyrosine phosphatase (PTP1B) and lipid phosphatases (SHIP2 and PTEN) effects are also considered in the model.

#### The TSC1/2-mTOR pathway

For the TSC1/2-mTOR pathway we mostly refer to the model recently published in Sonntag et al. [24], describing the mTOR effect in response to insulin and amino acids. The model considers both mTOR complexes: mTORC1 and mTORC2. mTORC1 activation is dependent on the presence of amino acids and is inhibited by the Tuberous sclerosis proteins 1 and 2 (TSC1/2) activation (i.e. Ser phosphorylation), which, in turn, depends on the 5′ AMP-activated protein kinase (AMPK) activation. AMPK activation depends on IRS1 Tyr phosphorylation, whereas TSC1/2 inhibition (i.e. Tyr phosphorylation) depends on AKT phosphorylation at Thr309. mTORC2, which was recently identified as the unknown phosphoinositide-dependent protein kinase 2 (PDK2), i.e. the kinase responsible for Ser474 phosphorylation of AKT [30–32], contributes to the double phosphorylation of AKT together with Thr309 phosphorylation, operated by the phosphoinositide-dependent protein kinase 1 (PDK1). Sonntag et al. [24] formulate the hypothesis of the presence of a PI3K variant, which is directly regulated by the activated insulin receptor and, in turn, activates mTORC2. We included this hypothesis in our model.

#### The RAS-MAPK pathway

For the RAS-MAPK pathway, we mostly refer to the model in Borisov et al. [26], describing both EGF and insulin stimulations. The model includes all the main chemical mechanisms involved in the RAS-MAPK pathway: the interaction of Tyr phosphorylated IRS1 with SHP2 (the SH2 domain-containing tyrosine protein kinase 2) and GRB2-SOS complex (the growth factor receptor-bound 2 and the son of sevenless complex), thus forming the SHP2-IRS1 and GRB2/SOS-IRS1 complexes, respectively; the GTP- binding of RAS; the phosphorylation of RAF proto-oncogene serine/threonine-protein kinase (c-RAF); the interaction of insulin receptor with Ras GTPase activating protein (RASGAP), which in turn catalyses the reverse process of Ras deactivation; the activation of the proto-oncogene tyrosine-protein kinase SRC which fully activates c-RAF; the dual specificity mitogen-activated kinase 1/2 (MEK1/2); the extracellular-signal-regulated kinases (ERK1/2).

#### Integration of the PI3K-AKT, the TSC1/2-mTOR and the RAS-MAPK pathways

The PI3K-AKT, TSC1/2-mTOR and RAS-MAPK pathways contain several overlapping parts, which, in the original papers, were often modelled in different ways being based on slightly different assumptions. We compared the overlapping reactions across different models and implemented the most up-to-date version of them based on the current knowledge of cellular biochemistry. Moreover, the integration of the three models required dealing with the different measurement units adopted to describe the state variables. Whereas immunoblot experiments permit to obtain important information concerning the timescale of signalling events, quantitative information about protein expression are often problematic to retrieve so that the predicted concentration profiles are sometimes reported in micromolar concentration, as in Borisov et al. [26], or in arbitrary units (AU), as in Sonntag et al. [24]. In contrast, in Sedaghat et al. [22], concentration were expressed either in molar units or in percentage of total concentration (e.g. GLUT4 cytosol concentration was considered to be 96 % of the total GLUT4 concentration in the cell at baseline condition).

Potentially, RBM allows performing both deterministic and stochastic simulations, provided that variables quantities are expressed in copies of molecules per cell. Even if in the present work we did not use stochastic simulation, we aligned all the variables to the same unit, i.e. number of molecules per cells, by multiplying the molar units by NA*V (NA indicates Avogadro number and V the cell volume, considered equal to 3e-12 l). All details about unit conversion from AU and percentage concentrations are given in Material and Methods.

The resulting model consists of 42 reaction rules and 101 parameters encoding the interactions among 61 distinct chemical species. Reactions, parameter values and initial conditions are all reported in the Additional file 1.

### Novelties of the RBM-ISP model

The RBM implementation, facilitated accounting for a number of the ISP features, such as the phosphorylation of signalling proteins at multiple sites and with multiple effects, the simultaneous interaction of molecules with different binding partners and the subcellular localization of some reactions. The above listed characteristics are discussed in details in the following.

#### Phosphorylation of signalling proteins on multiple sites

Signalling molecules may have different levels of activity, depending on which residues are phosphorylated. Consider for example IRS1 and AKT. IRS1 has many residues potentially involved in post-translational modifications and can be activated or inhibited in its kinase action, depending on the phosphorylated residue being Tyr or Ser [33,34]. For instance, Tyr-896 phosphorylation is required for PI3K, SHP2 and GRB2 binding, whereas Ser-636 phosphorylation by p70S6K is a mechanism related to insulin resistance [35]. AKT, in contrast, can be activated by phosphorylation at Thr309 or Ser474 by PDK1 and mTORC2, respectively [36].

IRS1 phosphorylation dependent activation/inhibition was already included in Sedeghat model although considering Ser phosphorylation regulated by PKC and not by p70S6K, as in Sonntag model. Here we modelled both PKC and p70S6K actions and described pIRS1-Tyr896 complex formation/dissociation with PI3K [22], SHP2 and GRB2 [26].

The two phosphorylation sites of AKT were not explicitly modelled in Sedaghat et al. [22]. We modelled the AKT phosphorylation at Thr mediated by PI(3,4,5)P_3_ as in Sedaghat et al. model and the AKT phosphorylation at Ser mediated by mTORC2 as in Sonntag et al. model and assumed:

i) AKT phosphorylated either at Thr or both sites to act on TSC1/2 complex by mediating its phosphorylation at Tyr and dephosphorylation at Ser [24];
ii) AKT phosphorylation at Ser or both sites to inactivate C-RAF [26];
iii) AKT phosphorylation at either Thr or Ser to activate GLUT4 translocation [22].

#### Interaction with multiple binding partners

The interaction of the molecules in the signalling pathways with numerous different binding partners results in the potential formation of different complexes. In ISP, IRS1 phosphorylated at Tyr-896 can bind PI3K as modelled in Sedaghat et al. [22] or GRB2/SOS and SHP2, as modelled in Borisov et al. [26]. In order to match the RBM-ISP model specification with the current knowledge, we allowed the formation of different complexes. Thus, IRS1 can bind in a mutually exclusive way GRB2/SOS and SHP2, but can bind simultaneously GRB2/SOS and the p85 regulatory subunit of PI3K [37,38].

#### Information about subcellular localization in reaction rules

The possibility that proteins have to interact is often related to their physical localization, i.e. their presence in the extra-cellular space, cytoplasm, nucleus, plasma membrane, etc. For instance, lipids PI(3,4,5)P_3_ function as plasma membrane docking sites that recruit different proteins containing pleckstrin homology (PH) domains (e.g. AKT and PDK1) and their co-localization can accelerate specific signalling events [39]. Another example is the interaction of mTORC1 with the Ras homologue enriched in brain (RHEB) and Rag family on lysosome membrane, reported by Zoncu et al. [16]. In our model, we included the information about subcellular localization for the insulin receptor and GLUT4 transporter, distinguishing between their plasmatic and cytoplasmic localization according to the mathematical description given by Sedaghat et al. [22].

### Model predictions

**Fig. 2:**
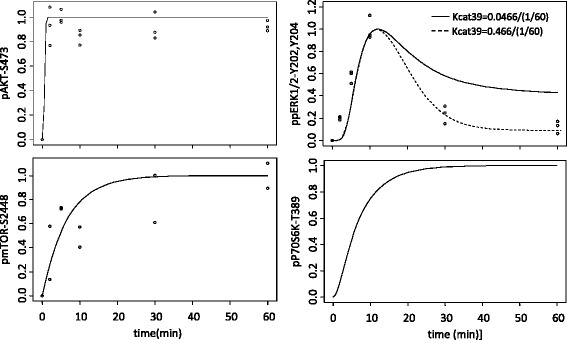
Comparison between simulated and experimental data. Comparison between experimental concentration (points) and normalized model predictions (lines) for pAkt-S473, ppERK1/2, pmTOR-S2448 and pP70S6K-T389. The profile of ppERK1/2-Y202,Y204 obtained by increasing the strength of the feedback between ERK and GRB2/SOS is shown in dotted lines. Values are reported for experimental data of human skeletal muscle cells (SkMCs) exposed to EBSS + 100 nM insulin at time 0′, 2′, 5′, 10′, 30′, and 60′. All measurements were taken in three biological replicated, and for each biological replicates, three technical replicated measurements were taken. All data are expressed in arbitrary units (AU) and rescaled between 0 and 1 for sake of comparison

The concentration profiles of all the chemical species populating the model were simulated upon 60 min, 100 nM insulin stimulation. The 100 nM concentration represents a well-accepted level of insulin stimulation in cell cultures commonly found in the literature [40] and used also by our group [41]. According to Sonntag et al. [24], we also assumed a constant amino acids stimulation, necessary to obtain the mTORC1 activation and the feedback of p70S6K on IRS1. To assess the model reliability, model predictions were compared with experimental data available in our dataset for some phosphoproteins at time 2, 5, 10, 30 and 60 min following insulin plus amino acids, i.e. leucine, stimulation [41]. As shown in Fig. 2, the experimental and predicted profiles of pAKT-S473 and pmTORC1-S2448 are in good agreement, since they both show an increasing phosphorylation pattern reaching a steady state in the first 2–5 min and 20–30 min, respectively. The predicted profile for ppERK1/2-Y202,Y204 is confirmed by experimental data in the first 10 min, whereas in the last time points it decreases rapidly in experimental data with respect to model predictions. The profile observed in experimental data for ppERK1/2-Y202,Y204, might be more closely matched by augmenting the strength of the feedback between ppERK1/2-Y202,Y204 and GRB2/SOS. In Fig. 2 (upper right panel, dotted line), the simulated profile of ppERK1/2-Y202,Y204 obtained by multiplying the parameter kcat39 (see Additional file 1) by a factor of 10 is shown. Note that in the RBM ISP model available online, we decided to leave the parameter of the model unchanged, i.e. we use the value from the literature [26], postponing parameter optimization for future studies, when further data will be available. Unfortunately, the experimental data of pP70S6K-T389 were not reliable (only one replicate available), so we cannot compare the experimental and simulated profiles for this protein, which is an endpoint of the pathway with an important feedback action on IRS1. Nevertheless, the simulated profile shown in Fig. 2 (lower, right panel) resembles experimental data shown in other papers [21].

**Fig. 3:**
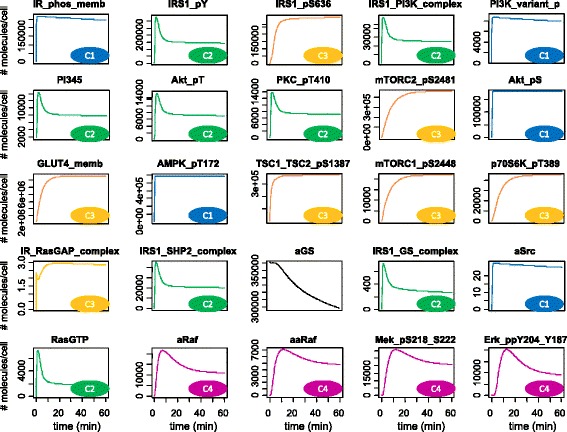
Clustering of simulated profiles. Four clusters were identified for the predicted profiles of the active species, according to their dynamic behaviour: 1) Fast response reaching the steady state within 2–5 min (blue); 2) Fast overshooting responses reaching a peak within 2–5 min and descending to a steady state after 10–20 min (green); 3) Slow response reaching the steady state in 10–20 min (orange); 4) Slow overshooting responses reaching a peak in 5–10 min and descending to a steady state in 30–60 min (purple)

We the examined the predicted profiles of all the active species and clustered them in four clusters according to their dynamic behaviour, as shown in Fig. 3:

1. Fast response, reaching the steady state within 2–5 min (blue)
2. Fast overshooting response, reaching the peak within 2–5 min and then the steady state after 10–20 min (green)
3. Slow response, reaching the steady state in 10–20 min (orange)
4. Slow overshooting responses, reaching the peak in 5–10 min and the steady state in 30–60 min (purple).

Following insulin stimulation, the insulin receptor responds rapidly by phosphorylating and triggering a cascade of events along distinct pathways. Along the PI3K-AKT pathway, all mechanisms tightly related to IRS1 Tyr- phosphorylation are characterized by a fast response. The same is true for other direct targets of IRS1 phosphorylated at Tyr along the TSC1/2-mTOR cascade, i.e. AMPK phosphorylation at T172, SHP2-IRS1 and GRB2/SOS-IRS1 complexes formation. The fast response is characterized by either a rapid rise to the steady state or by a transient overshoot followed by the steady state condition, depending on the absence/presence of feedback mechanisms acting on the target molecule (case 1 and 2, in blue and green, respectively, in Fig. 3). Mechanisms mostly constituting the TSC1/2-mTOR pathway and playing a role in Ser- phosphorylation of IRS1 are characterized by a relatively slow not-overshooting response (case 3, orange in Fig. 3). As observed above, the RAS-MAPK pathway assumes in its upstream components a rapid response, which becomes noticeably slower for the downstream ones (case 4, purple in Fig. 3).

In general, molecules downstream the pathway, related to relatively slow processes such as transcription activation or interaction with the environment outside the cell, such as ERK1/2 and GLUT4, are characterized by slow response whereas molecules upstream the pathway are characterized by a fast response so to elicit a rapid signal propagation. In this context, the overshooting response followed by the return to a steady state might help to achieve a rapid signal propagation on one signalling route, making molecules again available for other signalling routes immediately after.

### Model predictions in absence of control mechanisms

**Fig. 4:**
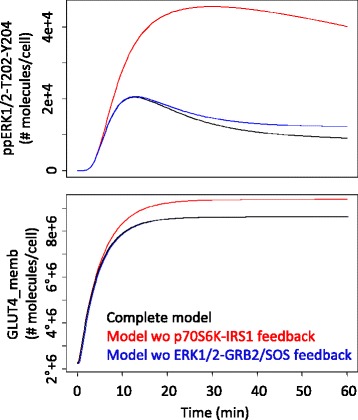
Simulated ppERK1/2-T202-Y204 (upper panel) and GLUT4 membrane concentration (lower panel) profiles upon 100 nM insulin stimulation, with the complete model (black), the model without p70S6K-IRS1 feedback (red) and the model without the ERK1/2-GS feedback (blue). This latter does not affect GLUT4 membrane concentration; therefore GLUT4 simulated profiles with and without the ERK1/2-GS feedback are superimposable

A number of simulations were then performed to investigate the robustness of the system on two target effects of ISP: GLUT4 translocation and ERK1/2 phosphorylation. In particular, the role of two major control mechanisms in regulating the target effects was analysed:

- P70S6K-IRS1 negative feedback loop (red line in Fig. 1);
- ERK1/2-GRB2/SOS negative feedback loop (blue line in Fig. 1);

To this purpose, the time course of GLUT4 and ERK1/2 response in presence and absence of the two regulatory mechanisms listed above was compared (Fig. 4). The dynamic of doubly phosphorylated ERK1/2 is strongly affected by both P70S6K-IRS1 and ERK1/2-GRB2/SOS negative feedback loops. On the other hand, the steady state and dynamic behaviour of GLUT4 in membrane are not affected by ERK1/2, but are strongly affected by P70S6K-IRS1 negative feedback loop, thus confirming the remarkable importance of this latter control mechanism in determining the system dynamics.

**Fig. 5:**
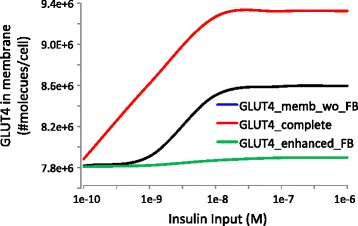
Simulated GLUT4 membrane concentration at 60 min upon different insulin stimulation, with the complete model (black), the model without p70S6K-IRS1 feedback (red) and the model with enhanced p70S6K-IRS1 feedback, obtained by increasing parameter k15 by 100 % of its vaue (green)

It is well known that insulin resistance is associated with defects in IRS-dependent signalling, implicating its dysregulation in the initiation and progression of metabolic disease. An emerging view is that the positive/negative regulation of IRS by autologous pathways is subverted in disease by increased basal and other temporally inappropriate serine/threonine phosphorylations [37], which lead to a reduced glucose uptake. Compensatory hyperinsulinaemia may rise at this point and lead, ultimately, to diabetes. Although IRS1 (and IRS2) are regulated through a complex mechanism involving phosphorylation of more than 50 different serine/threonine residues, in our model P70S6K-IRS1 negative feedback loop seems essential for a good control of glucose uptake. Enhancement of P70S6K-IRS1 negative feedback loop is able to explain a reduced insulin sensitivity and glucose uptake (Fig. 5). Similarly (although they also hypothesize a positive feedback from mTOR to a different IRS1 Serine residue), Brännmark et al. [21], using a minimal model of insulin signalling, show that a decreased positive feedback mechanism is able to explain a reduced glucose uptake.

On the other hand, both P70S6K-IRS1 and ERK1/2-GRB2/SOS negative feedback loops seem essential to guarantee that doubly phosphorylated ERK shows a transient behaviour, with a peak at 10 min followed by a return to the baseline condition. This behaviour has already been reported in the literature under insulin stimulus and under epidermal growth factor stimulus [42]. A transient ERK response prevents a sustained activation of ERK that would result in continual cell proliferation [42].

### Sensitivity analysis

To further investigate the role of the two major control mechanisms in regulating the target effects, local sensitivity analysis was performed by applying a small perturbation (0.1 % of parameter value) to one model parameter at a time and evaluating the resulting relative changes of GLUT4 translocation and ERK1/2 phosphorylation (see Methods). Tables 1 and 2 show the sensitivity coefficients of the model parameters, ranked accordingly to their absolute value and compared to the value the coefficients assume upon removal of P70S6K-IRS1 and ERK1/2-GRB2/SOS negative feedback loops. These coefficients measure the overall effect, i.e. during the observation window, of a parameter change on the outcome, i.e. GLUT4 and ERK response. Positive/negative values mean that increasing the parameter value has the effect of enhancing/reducing the response. Since small absolute values mean that the parameter changes do not significantly affect the outcome, in Tables 1 and 2, only coefficient greater than 0.1 % (absolute value), either in the original or modified model, are reported.

**Table 1:** Parametric sensitivity analysis of the complete model for GLUT4 membrane translocation

Parameter	Complete model	wo_feedback p70S6K	wo_feedback ERK1/2	Process
k_13	−84.89 %	−83.59 %	−84.89 %	GLUT4 translocation
k13p	66.02 %	66.76 %	66.02 %	GLUT4 translocation
k13	19.03 %	17.01 %	19.03 %	GLUT4 translocation
n_p70	−15.21 %	-	−15.21 %	p70 mediated IRS1 phosphorylation at Ser
k9a	8.25 %	13.87 %	8.25 %	lipids PI(3,4,5)P_3_ formation
k9s	−8.25 %	−13.86 %	−8.25 %	lipids PI(3,4,5)P_3_ formation
k8	7.36 %	10.88 %	7.36 %	IRS1_PI3K complex formation
k_8	−7.36 %	−10.87 %	−7.35 %	IRS1_PI3K complex formation
k_14	−5.25 %	−5.19 %	−5.25 %	GLUT4 degradation
k_7	−5.05 %	−2.68 %	−5.05 %	IRS1 dephosphorylation at Tyr
k7	5.05 %	2.68 %	5.05 %	IRS1 phosphorylation at Tyr
k_7p	4.45 %	0.53 %	4.45 %	IRS1 dephosphorylation at Ser
k15	−4.30 %	-	−4.30 %	IRS1 phosphorylation by P70S6K
Kd_p70	0.72 %	-	0.72 %	p70 mediated IRS1 phosphorylation at Ser
Vmax	0.65 %	2.18 %	0.65 %	PKC and p70 mediated IRS1 phosphorylation at Ser
k4p	−0.51 %	−0.27 %	−0.52 %	Phosphorylated receptor internalization
k_21	−0.18 %	-	−0.18 %	TSC1-TSC2 T1462_phosphorylation_by_Akt_pT309
k7p	−0.15 %	−0.54 %	−0.15 %	IRS1 phosphorylation at Ser
k41	0.14 %	0.46 %	0.14 %	IRS1-GS and IRS1-SHP2 complex disruption

Sensitivity coefficients are ranked accordingly to their absolute values and their corresponding values upon P70S6K-IRS1 and ERK1/2-GRB2/SOS negative feedback loop removal are also shown. Only coefficient greater than 0.1 % (absolute value) either in the original or modified model are reported. The column “Process” describes the biological process to which the parameter takes place

**Table 2:** Parametric sensitivity analysis of the complete model for ERK1/2 activation

Parameter	Complete model	wo_feedback P70S6K	wo_feedback ERK1/2	Process
n_p70	−433.62 %	-	−553.98 %	p70 mediated IRS1 phosphorylation at Ser
kcat33	158.53 %	159.60 %	207.34 %	# Mek phosphorylation
kcat32	−157.47 %	−158.57 %	−202.56 %	# Raf inactivation
k7	154.53 %	23.76 %	183.15 %	# IRS-1 phosphorylation at Tyr
k_7	−154.50 %	−23.96 %	−183.05 %	# IRS-1 dephosphorylation at Tyr
kcat28	152.95 %	65.18 %	189.32 %	Ras activation
kcat29	−152.78 %	−65.23 %	−188.99 %	# Raf activation
kcat30	150.90 %	62.38 %	185.97 %	# Raf activation
k_7p	140.15 %	4.82 %	161.27 %	# IRS-1 dephosphorylation at Ser
k15	−135.17 %	-	−155.79 %	# IRS1 phosphorylation by P70S6K
kcat35	79.97 %	79.91 %	104.72 %	Erk phosphorylation
kcat36	77.83 %	79.00 %	101.60 %	Erk phosphorylation
k41	−34.20 %	−16.98 %	−43.11 %	# IRS1-GS and IRS1-SHP2 complex disruption
kcat39	−23.10 %	−25.16 %	-	GS inhibition
Kd_p70	20.02 %	-	26.18 %	p70 mediated IRS1 phosphorylation at Ser
Vmax	15.35 %	19.63 %	21.95 %	PKC and p70 mediated IRS1 phosphorylation at Ser
k8	−13.39 %	−9.24 %	−17.15 %	# IRS-1_PI3-K complex formation (PI3-K activation)
k_8	13.38 %	9.27 %	17.04 %	# IRS-1_PI3-K complex dissociation
n	6.66 %	0.11 %	7.10 %	PKC mediated IRS1 phosphorylation at Ser
k_21	−5.65 %	-	−6.50 %	# TSC1-TSC2 T1462_phosphorylation_by_Akt_pT309
k9s	4.74 %	0.35 %	5.35 %	# lipids PI(3,4,5)P_3_ formation
k9a	−4.71 %	−0.28 %	−5.48 %	# lipids PI(3,4,5)P_3_ formation
k7p	−4.01 %	−4.79 %	−5.32 %	# IRS-1 phosphorylation at Ser
k4p	−2.62 %	−10.56 %	−4.01 %	# Phosphorylated receptor internalization
k_39	2.62 %	2.10 %	-	GS inactivation
k_20	2.20 %	-	−0.21 %	# p70S6K phosphorylation/dephosphorylation mediated by mTORC1_pS2448
kcat24	−1.71 %	87.60 %	17.49 %	Src activation
kcat31	−1.70 %	87.58 %	17.35 %	# Raf activation
alpha24	−1.60 %	87.59 %	17.48 %	Src activation
k_4	0.77 %	2.59 %	0.88 %	# Free receptor externalization
k_16	0.13 %	−0.07 %	−0.11 %	# AMPK_T172 dephosphorylation mediated by IRS1_pY
Kd_pkc	0.11 %	−0.02 %	0.00 %	PKC mediated IRS1 phosphorylation at Ser
k12	0.10 %	0.04 %	−0.03 %	# PKC phosphorylation at Threonine
k6	0.08 %	0.12 %	−0.01 %	# Receptor unbinding and dephosphorylation (inside the cell)
k_2	0.05 %	−0.11 %	−0.17 %	# Receptor binding 2nd insulin molecule

Sensitivity coefficients are ranked accordingly to their absolute values and their corresponding values upon P70S6K-IRS1 and ERK1/2-GRB2/SOS negative feedback loop removal are also shown. Only coefficient greater than 0.1 % (absolute value) either in the original or modified models are reported. The column “Process” describes the biological process to which the parameter takes place

Parametric sensitivity analysis of the complete model for GLUT4 response reveals that the most sensitive parameters are related to GLUT4 translocation to plasma membrane, followed by those related to lipid formation and IRS1_PI3K complex formation/dissociation. The absence of p70S6K_IRS1 feedback has a strong impact on augmenting the sensitivity (absolute value) of parameters related to lipid formation and IRS1_PI3K complex formation/dissociation.

Parameters related to baseline IRS1 phosphorylation/dephosphorylation at Tyr/Ser, have lower sensitivity, which diminishes (absolute value), in general, by removing P70S6K-IRS1 feedback, with the exception of parameter k7p, related to IRS1 phosphorylation at Ser. Parameters related to PKC mediated IRS1 phosphorylation at Ser also show increased values after removal of P70S6K-IRS1 feedback. Since ERK1/2-GRB2/SOS feedback removal has no effect on GLUT4 translocation, the sensitivity coefficients do not change with and without this feedback.

Parametric sensitivity analysis of the complete model for ERK1/2 response shows that the most sensitive parameters are related to RAS, MEK and RAF activation and to IRS1 phosphorylation at Tyr and Ser, this latter mediated by p70S6K.Along both the PI3K-AKT and the RAS-ERK1/2 pathway, ERK1/2-GRB2/SOS feedback seems to have an important role on the system robustness. The system dynamics are weakly affected by its absence (see Fig. 4), whereas the parameter sensitivity increases (in absolute value) in almost all cases if this feedback is removed.

Conclusions about the effect of P70S6K-IRS1 feedback on ERK1/2 response are more controversial. Removing this feedback has the effect of reducing parameter sensitivity along PI3K_AKT pathway, whereas, along RAS-ERK1/2 pathway, it has almost no effect for parameters related to MEK phosphorylation, RAF inactivation and ERK phosphorylation. It diminishes the sensitivity for parameters related to RAS, RAF activation and to IRS1-GRB2/SOS and IRS1-SHP2 complex disruption. It strongly augments the sensitivity of parameters related to SRC and RAF activation.

These results highlight the central role of negative feedback loops in determining not only the dynamics of a biological system but also its robustness. This property is of remarkable importance because it preserves the dynamic behaviour of the system against noise and small biological fluctuations commonly due to intercellular variability.

## Conclusions

We implemented a computational model of the ISP, including its most important regulatory mechanisms known at present. The model provides a comprehensive description of the system, since it integrates three previously published models [22,24,26] addressing distinct aspects of insulin signalling, such as the PI3K-AKT pathway, the TSC1/2-mTOR pathway, and the RAS-ERK1/2 pathway.

The model was implemented using the RBM approach, especially suitable for the specification of complex biochemical systems models, such as signalling networks. In particular, we made use of the BioNetGen software to provide a clear and compact representation of the chemical species and the reactions populating the system. RBM is based on the key assumption that molecular interactions are “modular”, meaning that the network dynamics is mostly determined by the local properties of the proteins involved in the interactions, and thus the same rate law can be assigned to a defined “class” of reactions. Hence, differently from the traditional approach in which a system is directly described by a number of ODEs equal to the number of possible interactions and transformations of chemical species, in the RBM approach the same system can be described using a reduced number of “rules”. In the case of the ISP model here presented, the dynamic of 61 distinct chemical species was encoded by only 42 rules.

The RBM-ISP model was partially validated using experimental data of some of the phosphorylated proteins involved in ISP such as pAKT-S473, ppERK1/2-T202-Y204, and pmTOR-S2448, measured at 0, 2, 5, 30 and 60 min after insulin stimulation. Despite some minor discrepancy at 60 min for ppERK1/2-T202-Y204, the experimental and predicted profiles are in good agreement.

As already noted in [41], since a wide range of signals, including nutrients, energy levels, growth factors, and amino acids are known to affect insulin signalling pathway, it is likely that experimental results strongly depend on the availability of combinations of the above variables. Moreover, the proteins involved in the insulin signalling pathway are known to exhibit a number of different phosphorylation sites, able to interact in different ways with different molecules, a complex set of regulatory mechanisms, which are not yet completely understood. In this respect, our model represents the state of the art knowledge of ISP and can be used together with experimental data as a useful simulation tool for the generation of new hypotheses. Moreover, since the RBM approach allows, thanks to its modularity, the easy integration of different pathways and the inclusion of new finer details as soon as they become available, our model might constitute a starting point model, ready for the inclusion of new regulatory mechanisms such as those regarding the regulation of IRS1 through its numerous phosphorylation sites [19,37]. Another example of regulatory mechanisms that might be integrated within our model in future studies, is given by Yugi et al. [43], in which the authors have reconstructed the insulin signal flow from phosphoproteome and metabolome data and developed a kinetic model of the glycolytic pathway.

Besides presenting the RBM-ISP model, in this work we showed one of its possible applications in the analysis of network robustness. Robustness is a fundamental property that permits to biological systems to preserve their specific functionality despite external and internal perturbations due, respectively, to unpredictable environmental changes and fluctuation of protein concentrations Kitano [44]. Biological robustness is largely related to the network topology and, in particular, to the presence of control mechanisms, such as positive and negative feedback loops [45, 46]. In this work, we investigated the role of two negative feedback loops involving P70S6K-IRS1 and ERK1/2-GRB2/SOS in controlling GLUT4 translocation and ERK1/2 phosphorylation, respectively. The removal of the P70S6K-IRS1 feedback showed relevant differences in the concentration profiles of both GLUT4 translocation to the cell membrane and ERK1/2 phosphorylation. Removal of the ERK1/2-GRB2/SOS feedback resulted in minor differences in ERK1/2 phosphorylation. However, the comparison between the original model and the model without the ERK1/2-GRB2/SOS feedback revealed that GLUT4 translocation and ERK1/2 phosphorylation are more sensitive to changes in the model parameters in the latter case. The reported results highlight how control mechanisms such as negative feedback loops may act in a concealed way, determining some of the fundamental network properties that might not be observed by simple inspection of the system dynamic behaviour, and stress on the need of more and more realistic models and computational tools, able to deal with large-scale simulations and interaction of smaller subnetwork models.

## Methods

### Initial concentration and unit conversion

We aligned all variables to the same unit, i.e. number of molecules per cells, by multiplying the molar units by NA*V (NA indicates Avogadro number and V the cell volume, considered equal to 3e-12 l).

To convert the AU concentration used in Sonntag model into number of molecules per cells we considered IRS1, which is present in both Sonntag and Borisov model as reference concentration, and scaled other quantities accordingly. So, for example, initial concentration of mTORC2, which was set to 18.8 AU in Sonntag et al., was multiplied by the initial concentration of IRS1 expressed in number of molecules per cells (as derived from [26]) and divided by IRS1 initial concentration expressed in AU.

As recently pointed out by Nyman et al. [47] the initial concentrations attributed to insulin receptor IR both at plasma membrane and in cytoplasm and to IRS1 and PIK3 molecules in the Sedaghat model, when multiplied by NA*V resulted in less than one molecule per cell. The initial concentrations of these four molecules were thus taken from the Sonntag model.

Finally, in absence of further information from the literature, concentrations expressed in percentage of the total concentration in Sedaghat model (namely, GLUT4, PKC, PI(3,4,5)P_3_, PI(3,4)P_2_ and PI(4,5)P_2_), were converted into number of molecules per cell by considering a total number of molecules equal to IRS1 for GLUT4 (in membrane plus cytoplasm) and total lipids (i.e. PI(3,4,5)P_3_ plus PI(3,4)P_2_ plus PI(4,5)P_2_) and a total number of molecules equal to AKT for PKC.

Initial concentrations are reported in Additional file 1: Table S1.

### Parameter values

Reaction rules and parameters (reported in Additional file 1) were taken from the original models and opportunely rescaled, when necessary, for sake of models integration accordingly to the changes made for the unit of measurements so to express parameters for first order reaction in 1/min and parameters for second order reactions in 1/(molecules*min).

The feedback of activated PKC and p70S6K on IRS1 and the modulatory effect of AKT on PTP, SHIP2 and PTEN, were modelled using Hill kinetics rather than linear kinetics for consistency among different models. Detailed functions and parameters are given in the Additional file 1.

### Experimental design

Muscle cell lines were stimulated with 100 nM insulin at time 0 for the following durations: 0′, 2′, 5′, 10′, 30′, and 60′. AKT, pAKT-S473, ERK1/2, ppERK1/2-T202-Y204, mTOR, pmTOR-S2448, P70S6K, and pP70S6K-T389 were measured using Western blot in technical triplicates. The experiment was repeated three times, thus three biological replicates are available for each condition.

### Cell cultures

Human skeletal muscle cells (SkMCs) and growth medium (SkGM) were purchased from Lonza. SkMCs are isolated from normal donors from gestational tissue usually from the quadriceps or psoas tissue and are sold in second passage. Cells were proliferated on 6-well plates between 5 and 7 passages at 37 °C, 5 % CO2, grown to 90 % confluence and exposed to differentiation medium (DMEM:F12 w/2 % Horse Serum and gentamycin) for ten days. Glucose was not added to the medium, but was present in the DMEM at 1000 mg/L. Cells were serum-starved with DMEM O/N and then switched to Earle’s Balanced Salt Solution (EBSS) for one hour, after which they were exposed to EBSS + 100 nM insulin. The 100 nM concentration represents a well-accepted level of insulin stimulation in cell cultures commonly found in the literature [40].

### Immunoblotting

Cells were lysed with Cell Signalling lysis buffer, sonicated, and centrifuged at 14KG for 30 min at 4 °C. Cell Signalling Lysis buffer was composed by 20 mM Tris–HCl (pH 7.5), 150 mM NaCl, 1 mM Na2EDTA, 1 mM EGTA, 1 % Triton, 2.5 mM sodium pyrophosphate, 1 mM bglycerophosphate, 1 mM Na3VO4, 1 μg/ml leupeptin, We also added Halt Protease and Phosphatase inhibitor cocktail. The Pierce 660 reagent was used to determine protein concentrations and lysates were loaded onto Invitrogen Nupage gels for Western blot transfer using the Biorad semi-dry Trans-blot apparatus. Blots were blocked with Licor blocking buffer and incubated overnight at 4 °C with target antibodies. Cell Signalling supplied all primary antibodies. In details, the antibodies used were: Akt total – Cell Signalling #2920; Akt Phospho (Ser473) – Cell Signalling #9336; ERK1/2 total – Cell Signalling #4695; ppERK1/2 (T202-Y204) – Cell Signalling #9106; Cell Signalling #9461; mTOR – Cell Signalling #2983; pmTOR (S2448) – Cell Signalling #2971; P70S6K – Cell Signalling #9202; pP70S6K (T389) – Cell Signalling #9206. All were used at a 1:1000 concentration.

Band densities were quantified using the Licor Odyssey system using the integrated intensity value for each band. To correct for the antibody efficiency, all band densities for each protein were expressed relative to the baseline time 0′. Moreover, to allow for comparison among different membranes, the ratios between phosphorylated and total proteins were calculated; total protein concentration is constant across time points as shown in [41]. Since for each protein, the total and the phosphorylated fractions were loaded in the same lane, this latter step also corrected for possible differences in the quantity of sample loaded in each gel. Finally, technical replicates were averaged.

### Computational modelling

The model of the insulin signalling pathway was implemented using BioNetGen [9], a software for the rule-based modelling.

To simulate the network dynamics, we first generated the network of all possible reactions that may occur in the system starting from the listed reaction rules and iteratively applied them to the set of seed species, and then performed a deterministic simulation through numerical solving of a system of ODEs.

### Parametric sensitivity analysis

For a generic prediction depending on *λ*_*1*_, *λ*_*2*_ … *λ*_*n*_ model parameters (and initial concentrations) and on time *t*:

$$ y(t)=f\left({\lambda}_1,{\lambda}_2,\dots {\lambda}_n,t\right) $$

the sensitivity of *y* at time *t* with respect to *λ*_*i*_ is defined as:

$$ {s}_i(t)=\frac{\partial f\left({\lambda}_1,\dots {\lambda}_i,\dots {\lambda}_n,t\right)}{\partial {\lambda}_i}\frac{\lambda_i}{y(t)} $$

and for each model parameters, the overall sensitivity can be derived as:

$$ {S}_i={\displaystyle \underset{t_0}{\overset{t}{\int }}}{s}_i\left(\tau \right)d\tau $$

and averaged across the observed time window [48, 49].

In order to implement the parametric sensitivity analysis, we exported our model in sbml language and ran the sensitivity analysis using COPASI [50].

## Additional files

Additional file 1: Model_details. Tables listing: (1) model inputs; (2) constant parameters; (3) the initial concentration of different chemical species; (4) the rules and functions used in the model, in BioNetGen syntax; (5) the model parameters. (DOCX 32 kb) Additional file 2: Protein_data. Experimental data of pAKT-S473, pmTORC1-S2448 and ppERK1/2-Y202,Y204 at time 2, 5, 10, 30 and 60 min following insulin plus amino acids, i.e. leucine, stimulation [41]. Data are available in three bioplogical replicates (A, B and C in the file). To correct for the antibody efficiency, all band densities for each protein are expressed relative to the baseline time 0′. Moreover, to allow for comparison among different membranes, the ratios between phosphorylated and total proteins are calculated. (XLSX 9 kb)

## Abbreviations

AKT: protein kinase B
AMPK: AMP-activated protein kinase
AU: arbitrary units
c-RAF: proto-oncogene serine/threonine-protein kinase
EGF: epidermal growth factor
ERK1/2: extracellular signal-regulated kinase 1/2
GLUT4: glucose transporter type 4
GRB2: growth factor receptor-bound 2
IR: insulin receptor
IRS1: insulin receptor substrate1
ISP: Insulin signalling pathway
MAPK: mitogen-activated protein kinase
MEK1/2: mitogen-activated kinase 1/2
mTOR: mammalian target of rapamycin
P70S6K: Ribosomal protein S6 kinase beta-1
PDK1: phosphoinositide-dependent protein kinase 1
PDK2: phosphoinositide-dependent protein kinase 2
PI(3,4)P2: phosphatidylinositol (3,4)-bisphosphate
PI(3,4,5)P3: phosphatidylinositol (3,4,5)-trisphosphate
PI(4,5)P2: phosphatidylinositol (4,5)-bisphosphate
PI3K: phosphatidylinositide 3-kinase
PKC: protein kinase C
PTEN: phosphatase and tensin homolog
PTP: protein tyrosine phosphatase
RBM: rule-based modeling
SHIP: phosphatidylinositol-3,4,5-trisphosphate 5-phosphatase 1
SHP2: SH2 domain-containing tyrosine protein kinase 2
SOS: son of sevenless complex
TSC1/2: tuberous sclerosis proteins 1 and 2
