# Supplementary material for: A rule-based model of insulin signalling pathway
Source: BMC Syst Biol. 2016 Jun 1;10:38. doi: 10.1186/s12918-016-0281-4 (PMC4888568; doi:10.1186/s12918-016-0281-4)
Supplement: Additional file 1: — Model_details. Tables listing: (1) model inputs; (2) constant parameters; (3) the initial concentration of different chemical species; (4) the rules and functions used in the model, in BioNetGen syntax; (5) the model parameters. (DOCX 32 kb) [file 12918_2016_281_MOESM1_ESM.docx]

**Table of inputs**

| **Constant** | **Meaning** | **Unit** |
| --- | --- | --- |
| I | Insulin input | Molecules |
| Amino_Acids | Amino acid input | Molecules |

**Table of constants**

| **Constant** | **Meaning** | **Value** | **Unit** |
| --- | --- | --- | --- |
| NA | Avogadro constant | 6.02E+23 | molecules |
| Vo | Volume outside the cell | 1.00E-10 | L |
| V | Volume inside the cell | 3.00E-12 | L |
| u | number of molecules/cell per nM concentration | NA*V*1e-9 | molecules |

**Table of Initial Concentrations**

The initial concentration of not reported species are assumed to be equal to 0

| **Molecule** | **# molecules/cell** | **Ref.** |
| --- | --- | --- |
| AKT_unphos | 180600 | 1 |
| PI3K | 361200 | 1 |
| IRS1_unphos | 541800 | 1 |
| IR_free_memb_init | 270900 | 1 |
| IR_free_cyt_init | 30100 | 1 |
| GRB2/SOS_active | 361200 | 1 |
| RasGAP | 90300 | 1 |
| SHP2 | 541800 | 1 |
| SRC_inactive | 935508 | 1 |
| RasGDP | 270900 | 1 |
| RAF_inactive | 180600 | 1 |
| MEK_unphos | 361200 | 1 |
| ERK_unphos | 722400 | 1 |
| AKT_unphos | 180600 | 1 |
| AMPK_unphos | 586920 | 2 |
| mTORC1­_unphos | 719538 | 2 |
| mTORC2_unphos | 537961 | 2 |
| p70S6K_unphos | 409311 | 2 |
| TSC1_TSC2_pT1462 | 426957 | 2 |
| PI3K_variant­_unphos | 541929 | 2 |
| PI(4,5)P_2_ | 538549 | 3 |
| PI(3,4,5)P_3_ | 1680 | 3 |
| PI(3,4)P_2_ | 1571 | 3 |
| PKC_unphos | 180600 | 3 |
| GLUT4_memb | 21672 | 3 |
| GLUT4_cyt | 520128 | 3 |

Ref: ^1^Borisov et al.(2009); ^2^Sonntag et al. (2012); ^3^Sonntag et al. (2002)

**Table of rules and functions (BioNetGen syntax)**

| **Reaction Rule** | **Function** | **Ref.** |
| --- | --- | --- |
| # Receptor binding 1st insulin molecule  IR(alpha,beta,Y999~u,NPXY,loc~m) + I(bs) <-> IR(alpha!1,beta,Y999~u,NPXY,loc~m).I(bs!1) k1,k_1 |  | 3 |
| # Receptor phosphorylation  IR(alpha!1,beta,Y999~u,NPXY,loc~m).I(bs!1) -> IR(alpha!1,beta,Y999~p,NPXY,loc~m).I(bs!1) k3 |  | 3 |
| # Receptor binding 2nd insulin molecule  IR(alpha!1,beta,Y999~p,NPXY,loc~m).I(bs!1) + I(bs) <-> IR(alpha!1,beta!2,Y999~p,NPXY,loc~m).I(bs!1).I(bs!2) k2,k_2 |  | 3 |
| # Receptor unbinding and dephosphorylation (on the cell membrane)  IR(alpha!1,beta,Y999~p,NPXY,loc~m).I(bs!1) -> IR(alpha,beta,Y999~u,NPXY,loc~m) + I(bs) f_3() | f3()=(k_3)*PTP*(1-Vmax/2*(Akt_pT)^n/(Kd_akt+(Akt_pT)^n)) | 3 |
| # Free receptor internalization/externalization  IR(alpha,beta,Y999~u,NPXY,loc~m) <-> IR(alpha,beta,Y999~u,NPXY,loc~c) k4, k_4 |  | 3 |
| # Phosphorylated receptor internalization/externalization  IR(alpha!+,Y999~p,NPXY,loc~m) <-> IR(alpha!+,Y999~p,NPXY,loc~c) k4p, k_4p |  | 3 |
| # Receptor synthesis  SynthIR() -> SynthIR() + IR(alpha,beta,Y999~u,NPXY,loc~c) k5 |  | 3 |
| # Receptor degradation  IR(alpha,beta,Y999~u,NPXY,loc~c) -> Degr() k_5 |  | 3 |
| # Receptor unbinding and dephosphorylation (inside the cell)  IR(alpha!+,Y999~p,NPXY,loc~c) -> IR(alpha,beta,Y999~u,NPXY,loc~c) f6() | f6()=(k6)*PTP*(1-Vmax/2*(Akt_pT)^n/(Kd_akt+(Akt_pT)^n)) | 3 |
| # IRS-1 phosphorylation/dephosphorylation in Tyr  IRS1(YXXM,Y896,Y~u,S636~u) <-> IRS1(YXXM,Y896,Y~p,S636~u) f7(),f_7() | f7()=k7*IR_phos_memb/IR_free_memb  (k_7)*PTP*  (1-Vmax/2*(Akt_pT)^n/(Kd_akt+(Akt_pT)^n)) | 3 |
| # IRS-1 phosphorylation/dephosphorylation in Ser  IRS1(YXXM,Y896,Y~u,S636~u) <-> IRS1(YXXM,Y896,Y~u,S636~p) f7p(), k_7p | f7p()=k7p*Vmax*(PKC_pT410)^n/  (Kd_pkc+(PKC_pT410)^n) | 3 |
| # IRS-1_PI3-K complex formation (PI3-K activation)  IRS1(YXXM,Y896,Y~p,S636~u) + PI3K(SH2) <-> IRS1(YXXM!1,Y896,Y~p,S636~u).PI3K(SH2!1) k8,k_8 |  | 3 |
| # lipids formation  PI45() <-> PI345() f9(),k_9*PTEN  PI34() <-> PI345() k10,k_10*SHIP | f9()=k9a*IRS1_PI3K_complex+k9b | 3 |
| # Akt phosphorylation/dephosphorylation in Tyr  Akt(T309~u) <-> Akt(T309~p) f11(),k_11 | f11()=k11*(PI345-PI345)/(PI345*10-PI345) | 3 |
| # PKC phosphorylation /dephosphorylation  PKC(T410~u) <-> PKC(T410~p) f12(), k_12 | f12()=k12*(PI345-PI345)/(PI345*10-PI345) | 3 |
| # GLUT4 translocation  GLUT4(loc~c) <-> GLUT4(loc~m) f13(), k_13 | f13()=k13+k13p*((0.2*(Akt_pT309+Akt_pS474+Akt_pp)/Akt)+(0.8*PKC_pT410/PKC)) | 3 |
| # GLUT4 synthesis  SynthG4() -> SynthG4() + GLUT4(loc~c) k14 |  | 3 |
| # GLUT4 degradation  GLUT4(loc~c) -> Degr() k_14 |  | 3 |
| # PI3K_variant phosphorylation/dephosphorylation by IRp  PI3K_variant(Y~u) <-> PI3K_variant(Y~p) fp15(),p_15 | fp15()=p15*IR_phos_memb | 2 |
| # IRS1 phosphorylation by P70S6K  IRS1(YXXM,Y896,Y~u,S636~u) + p70S6K(T389~p) -> IRS1(YXXM,Y896,Y~u,S636~p) + p70S6K(T389~p) f15() | f15()=k15*(Vmax)*(p70S6K_pT389)^n_p70/(Kd_p70+(p70S6K_pT389)^n_p70) | 2 |
| # AMPK_T172_phosphorylation/dephosphorylation mediated by IRS1_pY  AMPK(T172~u) <-> AMPK(T172~p) f16(),k_16 | f16()=k16*IRS1_pY | 2 |
| # Akt phosphorylation/dephosphorylation in Ser mediated by mTORC2_pS2481  Akt(S474~u) <-> Akt(S474~p) f17(),k_17 | f17()=k17*mTORC2_pS2481 | 2 |
| # TSC1-TSC2 S1387_phosphorylation_by_AMPK_pT172 and T1462_phosphorylation_by_Akt_pT308  TSC1_TSC2(T1462~p,S1387~u) <-> TSC1_TSC2(T1462~u,S1387~p) f21(),f_21() | f_21()=k_21*Akt_pT  f21()=k21*AMPK_pT172 | 2 |
| # mTORC1 phosphorylation/dephosphorylation mediated by TSC1_TSC2_pS1387 and Aminoacids  mTORC1(S2448~p) <-> mTORC1(S2448~u) f_18(),f18() | f_18()=k_18*TSC1_TSC2_pS1387  f18()=k18*Aminoacids | 2 |
| # mTORC2 phosphorylation/dephosphorylation mediated by phosphorylated PI3K_variant  mTORC2(S2481~u) <-> mTORC2(S2481~p) f19(),k_19 | f19()=k19*PI3K_variant_p | 2 |
| # p70S6K phosphorylation/dephosphorylation mediated by mTORC1_pS2448  p70S6K(T389~u) <-> p70S6K(T389~p) f20(),k_20 | f20()=k20*mTORC1_pS2448 | 2 |
| # RasGAP module  IR(Y999~p,NPXY,loc~m) + RasGAP(bs) <-> IR(Y999~p,NPXY!1,loc~m).RasGAP(bs!1) k22,k_22 |  | 1 |
| # Src module  Src(state~i) <-> Src(state~a) f24(), f25() | f24()=(0.1/10)*kcat24*(alpha24*IR_phos_memb)/(Km24+iSrc)  f25()=V25/(Km25+aSrc) | 1 |
| # IRS1-GS complex formation |  | 1 |
| IRS1(Y896,Y~p,S636~u) + GS(SH2,state~a) <-> IRS1(Y896!1,Y~p,S636~u).GS(SH2!1,state~a) k26,k_26 |  | 1 |
| # IRS1-SHP2 complex formation  IRS1(Y896,Y~p,S636~u) + SHP2(SH2) <-> IRS1(Y896!1,Y~p,S636~u).SHP2(SH2!1) k27,k_27 |  | 1 |
| # Ras module  Ras(state~GDP) <-> Ras(state~GTP) f28(), f29() | f28()=kcat28*(IRS1_GS_complex)/(Km28+RasGDP)  f29()=kcat29*(bRasGAP+IR_RasGAP_complex)/(Km29+RasGTP) | 1 |
| # Raf module  Raf(state~i) -> Raf(state~a) f30()  Raf(state~a) -> Raf(state~aa) f31()  Raf(state~aa) -> Raf(state~i) f32() | f30()=kcat30*RasGTP/(Km30+Raf)  f31()=kcat31*aSrc/(Km31+aRaf)  f32()=kcat32*PKA/(Km32+aaRaf)+alpha32*(Akt_pT309+beta32*Akt_pp) | 1 |
| # Mek module  Mek(S218_S222~u) <-> Mek(S218_S222~p) f33(), f34() | f33()=kcat33*aaRaf/(Km33+Mek)  f34()=V34/(Km34+Mek_pS218_S222) | 1 |
| # ERK module  Erk(T202_T185~u,Y204_Y187~u) <-> Erk(T202_T185~p,Y204_Y187~u) f35(), f38()  Erk(T202_T185~p,Y204_Y187~u) <-> Erk(T202_T185~p,Y204_Y187~p) f36(), f37() | f35()=kcat35*Mek_pS218_S222/(Km35+Erk+Erk_pT202_T185*Km35/Km36)  f36()=kcat36*Mek_pS218_S222/(Km36+Erk_pT202_T185+Erk*Km36/Km35)  f37()=V37/(Km37+Erk_ppY204_Y187+Erk_pT202_T185*Km37/Km38)  f38()=V38/(Km38+Erk_pT202_T185+Erk_ppY204_Y187*Km38/Km37) | 1 |
| # GS inhibition  GS(SH2,state~a) <-> GS(SH2,state~i) f39(),f_39() | f39()=kcat39*Erk_ppY204_Y187/(Km39+GS)  f_39()=k_39 | 1 |
| # IRS1-GS and IRS1-SHP2 complex disruption and IRS1 dephosphorylation  IRS1(YXXM,Y896!1,Y~p,S636~u).GS(SH2!1,state~a) -> IRS1(YXXM,Y896,Y~u,S636~u) + GS(SH2,state~a) f40()  IRS1(YXXM,Y896!1,Y~p,S636~u).SHP2(SH2!1) -> IRS1(YXXM,Y896,Y~u,S636~u) + SHP2(SH2) k41 | f40()=V40/(Km40+IRS1_GS_complex) | 1 |
| # SHP2 activity against RasGAP  IR(Y999~p,NPXY!1,loc~m).RasGAP(bs!1) -> IR(Y999~p,NPXY,loc~m) + RasGAP(bs) f42() | f42()=k42*IRS1_SHP2_complex | 1 |

Ref: ^1^Borisov et al.(2009); ^2^Sonntag et al. (2012); ^3^Sonntag et al. (2002)

**Table of Parameters (Rate Constants)**

| **Parameter** | **Value** | **Unit** |
| --- | --- | --- |
| k1 | (6e7/(NA*Vo)) | 1/(molecules*min) |
| k_1 | 0.2 | 1/min |
| k2 | (6e7/(NA*Vo)) | 1/(molecules*min) |
| k_2 | 20 | 1/min |
| k3 | 2500 | 1/min |
| k_3 | 0.2 | 1/min |
| PTP | 1 | - |
| PTEN | 1 | - |
| SHIP | 1 | - |
| n | 4 | - |
| n_p70 | 2 | - |
| Kd_akt | 2.955e12 | molecules^n |
| Kd_pkc | 2.955e12 | molecules^n |
| Kd_p70 | 4e8 | molecules^n_p70 |
| k4 | 0.003/9 | 1/min |
| k_4 | 0.003 | 1/min |
| k4p | 2.10E-03 | 1/min |
| k_4p | 2.10E-04 | 1/min |
| k_5 | ln(2)/(9*60) | 1/min |
| k5 | 38.63653729 | molecules/min |
| k6 | 0.461 | 1/min |
| k7 | 4.16 | 1/min |
| k_7 | 2.22 | 1/min |
| k7p | ln(2)/2 | 1/min |
| k_7p | 1.386 | 1/min |
| k8 | 5.18091E-06 | 1/(molecules*min) |
| k_8 | 10 | 1/min |
| k9s | ln(2)*2 | 1/min |
| k_9 | (94/3.1)*k9s | 1/min |
| k9b | (0.31/99.4)*k_9 | 1/min |
| k9a | 1.99817E-05 | 1/min |
| k_10 | ln(2)*4 | 1/min |
| k10 | (3.1/2.9)*k_10 | 1/min |
| k11 | ln(2)/2 | 1/min |
| k_11 | 10*k11 | 1/min |
| k12 | ln(2)/2 | 1/min |
| k_12 | 10*k12 | 1/min |
| k13 | (4/96)*0.167 | 1/min |
| k_13 | 0.167 | 1/min |
| k13p | ((40/60)-(4/96))*0.167 | 1/min |
| k14 | 0.001155*GLUT4_cyt | molecules/min |
| k_14 | 0.001155 | 1/min |
| p15 | 6.113E-07 | 1/min |
| p_15 | 10 | 1/min |
| k15 | 12 | 1/min |
| k16 | 3.423E-04 | 1/min |
| k_16 | 1.072E-02 | 1/min |
| k_17 | 3.415E-02 | 1/min |
| k17 | 4.592E-04 | 1/min |
| k_18 | 3.726E-07 | 1/min |
| k18 | 1.481E-08 | 1/min |
| k_19 | 1.837E-02 | 1/min |
| k19 | 1.311E-05 | 1/min |
| k20 | 3.927E-06 | 1/min |
| k_20 | 6.931E-01 | 1/min |
| k21 | 1.277E-06 | 1/min |
| k_21 | 1.765E-05 | 1/min |
| k22 | 6.66e-8/(u*1/60) | 1/(molecules*min) |
| k_22 | 2e6*k22*u | 1/min |
| kcat24 | 6.66/(1/60) | 1/min |
| alpha24 | 2.50E-04 | - |
| Km24 | 110*u | molecules |
| V25 | 6.66*u/(1/60) | molecules/min |
| Km25 | 50*u | molecules |
| k26 | 6.66e-4/(u*1/60) | 1/(molecules*min) |
| k_26 | 1e5*k26*u | 1/min |
| k27 | 6.66e-4/(u*1/60) | 1/(molecules*min) |
| k_27 | 1000*u*k27 | 1/min |
| kcat28 | 5.33/(1/60) | 1/min |
| Km28 | 50*u | molecules |
| bRasGAP | 1e-5*u | molecules |
| kcat29 | 2e4/(1/60) | 1/min |
| Km29 | 50*u | molecules |
| kcat30 | 0.1/(1/60) | 1/min |
| Km30 | 400*u | molecules |
| kcat31 | 3.33/(1/60) | 1/min |
| Km31 | 10*u | molecules |
| kcat32 | 0.666/(1/60) | 1/min |
| PKA | 100*u | molecules |
| Km32 | 1e4*u | molecules |
| alpha32 | (1e-6)/(u*(1/60)) | 1/(molecules*min) |
| beta32 | 2 | - |
| kcat33 | 0.133/(1/60) | 1/min |
| Km33 | 50*u | molecules |
| V34 | 16.6*u/(1/60) | molecules/min |
| Km34 | 675.299*u | molecules |
| kcat35 | 0.333/(1/60) | 1/min |
| Km35 | 500*u | molecules |
| Km36 | 500*u | molecules |
| kcat36 | 0.666/(1/60) | 1/min |
| V37 | 33.3*u/(1/60) | molecules/min |
| Km37 | 500*u | molecules |
| Km38 | 500*u | molecules |
| V38 | 23.33*u/(1/60) | molecules/min |
| kcat39 | 0.0466/(1/60) | 1/min |
| Km39 | 5000*u | molecules |
| k_39 | 6.66e-5/(1/60) | 1/min |
| V40 | 333*u/(1/60) | molecules/min |
| Km40 | 143.3*u | molecules |
| k41 | 0.666/(1/60) | 1/min |
| k42 | 0.0133/(u*1/60) | 1/(molecules*min) |
